# Altered neuronatin expression in the rat dorsal root ganglion after sciatic nerve transection

**DOI:** 10.1186/1423-0127-17-41

**Published:** 2010-05-28

**Authors:** Kuan-Hung Chen, Chien-Hui Yang, Jiin-Tsuey Cheng, Chih-Hsien Wu, Wei-Dih Sy, Chung-Ren Lin

**Affiliations:** 1 Department of Anesthesiology, Chang Gung Memorial Hospital-Kaohsiung Medical Center, Chang Gung University College of Medicine, Kaohsiung, Taiwan; 2 Department of Biological Sciences, National Sun Yat-Sen University, Kaohsiung, Taiwan; 3 Department of Anesthesiology, National Taiwan University College of Medicine, Taipei, Taiwan

## Abstract

**Background:**

Several molecular changes occur following axotomy, such as gene up-regulation and down-regulation. In our previous study using Affymetrix arrays, it was found that after the axotomy of sciatic nerve, there were many novel genes with significant expression changes. Among them, neuronatin (Nnat) was the one which expression was significantly up-regulated. Nnat was identified as a gene selectively expressed in neonatal brains and markedly reduced in adult brains. The present study investigated whether the expression of Nnat correlates with symptoms of neuropathic pain in adult rats with transected sciatic nerve.

**Methods:**

Western blotting, immunohistochemistry, and the Randall and Selitto test were used to study the protein content, and subcellular localization of Nnat in correlation with pain-related animal behavior.

**Results:**

It was found that after nerve injury, the expression of Nnat was increased in total protein extracts. Unmyelinated C-fiber and thinly myelinated A-δ fiber in adult dorsal root ganglions (DRGs) were the principal sub-population of primary afferent neurons with distributed Nnat. The increased expression of Nnat and its subcellular localization were related to mechanical hyperalgesia.

**Conclusions:**

The results indicated that there was significant correlation between mechanical hyperalgesia in axotomy of sciatic nerve and the increased expression of Nnat in C-fiber and A-δ fiber of adult DRG neurons.

## Background

Neuropathic pain in humans can be caused by peripheral nerve injury resulting in spontaneous pain, thermal and mechanical hyperalgesia and allodynia. Such clinical symptoms and signs frequently affect the subject's quality of life and functional status. It may even lead to psycho-social incapacitation.

Neuropathic pain still remains refractory to treatment, as available clinical therapies are often inadequate. In order to better understand the underlying molecular mechanisms of neuropathic pain and to develop new effective therapies, numerous research studies have focused on molecular changes after peripheral nerve injury that may participate in generation and maintenance of neuropathic pain. Our previous study using Affymetrix arrays (Additional file 1) identified many novel genes with significant expression changes, and one of them was neuronatin (Nnat).

Nnat was initially recognized as a gene selectively expressed in neonatal brains and markedly reduced in brains from young and aged adults. It was therefore thought to be involved mainly in neuronal cell growth and differentiation during brain development [1]. Nnat was also identified as an imprinting gene, and has two alternative mRNA spliced forms: neuronatin-α (Nnat-α) and neuronatin-β (Nnat-β). Both have the same open reading frame with Nnat-α encoding 81 aa and Nnat-β 54 aa. It has been suggested that Nnat-α has three exons and Nnat-β contains only the first and third exons. Nnat-β therefore appears to be derived from the α-form with the middle exon spliced out [2].

To date, no study has examined the altered expression of Nnat in dorsal root ganglion (DRG) after axotomy of sciatic nerve in adult rats. This research study was therefore conducted using Western blot, immunohistochemical studies, and animal behavior test to establish the possible role of Nnat in DRG after peripheral nerve injury.

## Methods

The experimental protocol was reviewed and approved by the Institutional Animal Care and Use Committee of the Chang Gung Memorial Hospital. The male Sprague-Dawley (S-D) rats were housed in solid floored cages with a deep layer of sawdust and cages were changed daily. A 12:12 h light-dark cycle was used and animals were allowed free access to sufficient food and water. A peripheral nerve axotomized model was used to generate neuropathic pain [3]. Approximately 8 weeks old male S-D rats (*n *= 36) weighing 270-300 gram received a unilateral (left) sciatic nerve transection under deep anesthesia with inhaled anesthetic and oxygen (2% Isoflurane with 2 L/min oxygen flow). The left sciatic nerve was exposed via a lateral approach. Proximal to the sciatic trifurcation, the nerve was freed of adhering connective tissue along 7-10 mm. One third to one half of the nerve was ligated tightly with 4-O chromic gut suture then the nerve was transected distal to the ligation. The incision was then closed in layers under aseptic conditions. The control (sham-operation; *n *= 9) rats underwent the same surgical procedure as described above but without nerve ligation and transaction. Finally, the animals were sacrificed at the following time points: post-operation day 1, 3, 5, and 7 (*n *= 9 for each group). Ipsilateral (left) injured L4-L5 DRGs were taken as specimens for further molecular and biochemical assessment.

The rat paw pressure test [4] was used for determination of hyperalgesia in male S-D rats of approximately 8 weeks of age (*n *= 10 for control and each time group). An Ugo-Basile pressure apparatus was used to assess pressure pain thresholds prior to sciatic nerve transection and again at different times. Testing was blind in regard to group designation. Brief, increasing force (16 g/s) was applied to the right hind paw. The force needed to induce paw withdrawal was recorded as the pain threshold. To reduce stress, the rats were familiarized with the testing procedure the day before the experiment. To minimize damage to the paw, a maximum force of 160 g (i.e. cut-off) was established for the test applied after the pharmacological treatments.

For the Western blot study, left L4-L5 DRGs from 3 rats of control and experimental group (post-axotomy day 1, 3, 5, and 7) were pooled and homogenized in lysis buffer. Protease inhibitor (Sigma) was added to prevent protein degradation. The protein content of supernatant was determined by BCA Protein Assay (Pierce, USA). Total Nnat (Nnat-α and Nnat-β) and glyceraldehydes 3-phosphate dehydrogenase (GAPDH) was electrophoretically separated on 10% SDS-polyacrylamide gel at 120 volts for 90 min. Proteins were then transferred to the polyvinylidene difluoride (PVDF) membrane (Immobilon-P, Millipore, 0.45 μM pore size) in a transfer buffer at 100 volts for 60 min at 4°C. The membrane was blocked overnight at 4°C with 5% non-fat dry milk in Tris-buffered saline Tween-20 (TBST: 20 mM Tris-HCl, 137 mM NaCl, 0.1% Tween-20, pH 7.5). The PVDF membrane was incubate with rabbit polyclonal antibody specific for total Nnat (Nnat-α and Nnat-β) (1:300; Santa Cruz, USA) and mouse monoclonal antibody specific for GAPDH (1:7500; Santa Cruz, USA) for 16-18 hours at 4°C. Blots were washed with TBST first and then incubated with goat anti-rabbit IgG and goat anti-mouse IgG horseradish peroxidase-conjugated secondary antibody (1:10000 for goat anti-rabbit IgG and 1:5000 for goat anti-mouse IgG; Santa Cruz, USA) for 1 hour at room temperature. Color molecular weight standard markers were run on each gel. Western blot results were quantified by densitometry.

For immunohistochemical studies, left L4-L5 DRGs were removed from the experimental (axotomized) and control (sham) groups and post-fixed for 2-3 h in 4% paraformaldehyde in 0.1 M phosphate buffer (PBS). The fixed DRGs were then cryoprotected, cut on a cryostat at 10 μm thickness and mounted serially onto Superfrost/plus microscope slides (Fisher Scientific) for staining. After incubation in 0.3% Triton X-100 in PBS (PBST) for 2 h, the sections were blocked in 10% goat serum in PBST for at least 4 h, followed by incubation with the primary antibody overnight at 4°C. The following primary antibodies were used: mouse monoclonal anti-Nnat (1:400; Santa Cruz, USA), rabbit polyclonal anti-NF-200 (1:1000; Sigma, USA), and rabbit polyclonal anti-Peripherin (1:1000; Sigma, USA). After being washed three times with PBST, the sections were incubated with secondary antibodies at following concentrations: Alexa fluor 488 goat anti-mouse IgG (1:1000; Invitrogen, USA), and Alexa fluor 594 goat anti-rabbit IgG (1:1000; Invitrogen, USA). The sections were then washed and cover slipped using ProLong™ Antifade mountant (Molecular Probes). The sections were examined in a confocal fluorescence microscope (Leica, TCS, SPII, Germany) and images captured with a Nikon CoolPix 990 camera (Nikon, Tokyo, Japan). Figures were assembled in Photoshop™.

All experimental data were presented as mean ± SEM and carried out with one-way ANOVA with Tukey's post-hoc analysis. A *P *value of < 0.05 was considered significant.

## Results

**Figure 1:**
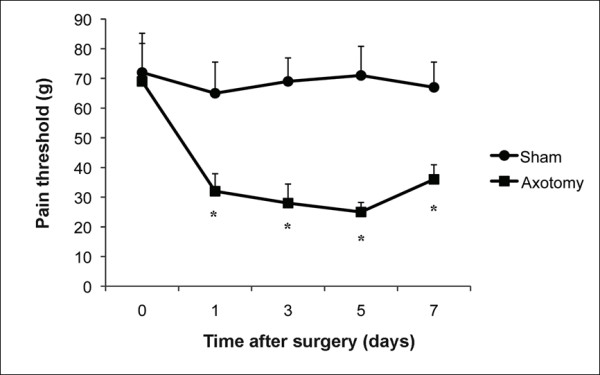
**Evaluation of hyperalgesia induced by rat sciatic nerve transection (neurectomy)**. Pain threshold was estimated in the rat paw pressure test applied before (time 0), and on days (d) 1, 3, 5, and 7 after surgery in male S-D rats of approximately 8 weeks of age. Sham-operated (Sham) rats were subjected to the same surgical procedure, without manipulation of the nerve. Time 0 corresponds to pain threshold measure before surgery. Data represent mean values ± S.E.M. for 10 rats per group. There was significant difference to the values obtained before surgery (time 0). There was also significant difference to the values of Sham group (* *P *< 0.05).

The sciatic nerve transection caused a significant decrease in pain threshold detected 2 h after surgery, as measured by the Randall and Selitto test. This phenomenon was detected up to 7 days after surgery (Figure 1). Sham-operated (control) animals did not present an alteration in pain threshold compared to basal values obtained immediately before the surgical procedure (Figure 1).

**Figure 2:**
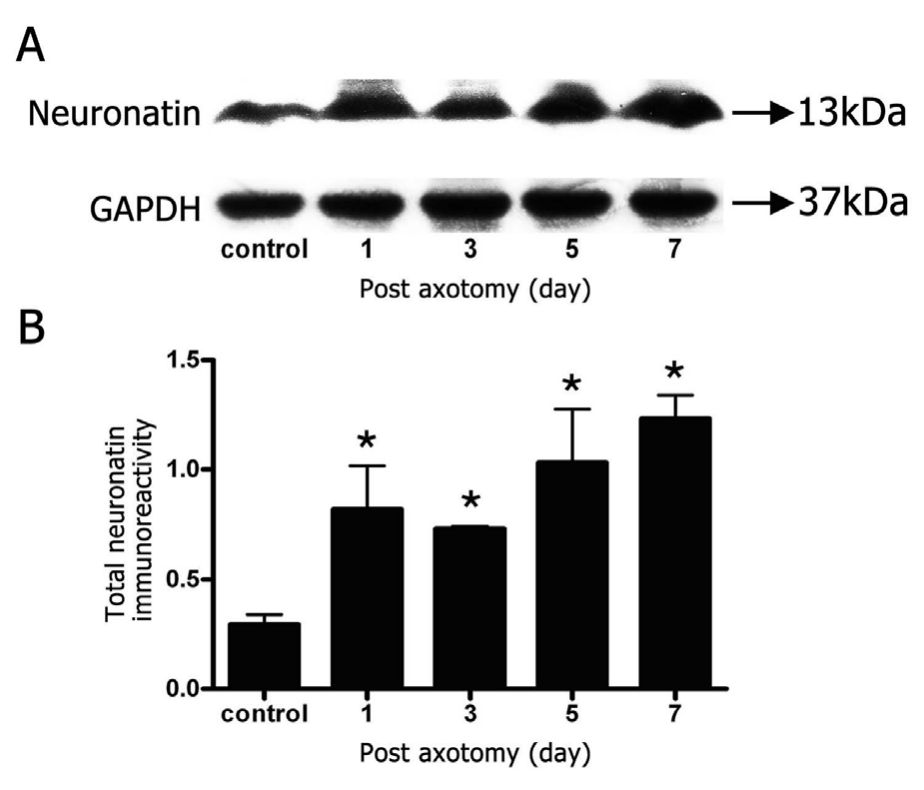
**Total neuronatin protein contents (Nnat-α and Nnat-β) at different time points after axotomy of sciatic nerve in adult rats**. (A) The total neuronatin protein contents from DRGs of control and post-axotomy day 1, 3, 5, and 7 were subjected to Western blot analysis. (B) The total neuronatin protein expression levels. Individual band was quantitated, and normalized with the density of its own GAPDH. Value are presented as mean ± S.E.M. (* *P *< 0.05 compared with control rats, *n *= 9 for each group).

To investigate whether the increased expression of Nnat found by our previous work with Affymetrix arrays was consistent with increased protein expression, left L4-L5 DRGs from 3 rats of control and experimental group (post-axotomy day 1, 3, 5, and 7) were pooled for Western blotting in triplicate (*n *= 9 for each group) to analyze total Nnat (Nnat-α and Nnat-β) expression level. As shown in Figure 2A, the expression level of total Nnat (a molecular weight band of ~13 kDa) was progressively up-regulated at 1, 3, 5, and 7 days post-axotomy compared to the sham control rats. To quantify the band of each time point, individual bands were normalized with the density of GAPDH bands. As shown in Figure 2B, the immunoreactivity of total Nnat displayed significant up-regulation compared with the sham control rats. The signal changes of total Nnat were expressed as 2.8, 2.5, 3.5, and 4.2 times at time points 1, 3, 5, and 7 days post-axotomy respectively compared to control rats (*P *< 0.05, *n *= 9 for each group).

**Figure 3:**
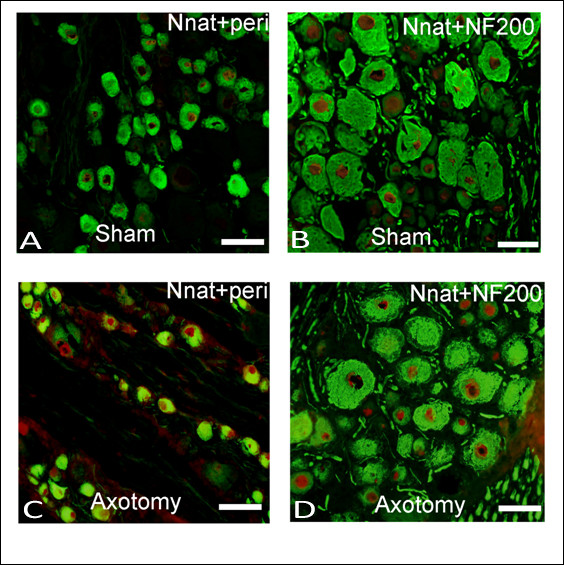
**Immunohistochemistry of neuronatin, NF200 (marker for large myelinated A-β fiber neurons), and peripherin (marker for unmyelinated C-fiber and thinly myelinated A-δ fiber neurons) in DRG neurons on post-axotomy day 7**. Confocal image of DRG neurons immunostained with anti-neuronatin (red), anti-NF200 (green), and anti-peripherin (green). The double immunoreactivity-positive DRG neurons for neuronatin and peripherin (A) or neuronatin and NF200 (B) in control sham group, and neuronatin and peripherin (C) or neuronatin and NF200 (D) in post-axotomy day 7 group are illustrated. Note that the neurons that were double immunoreactive for Nnat and peripherin were increased in axotomized rats. Scale bar, 50 μm.

**Figure 4:**
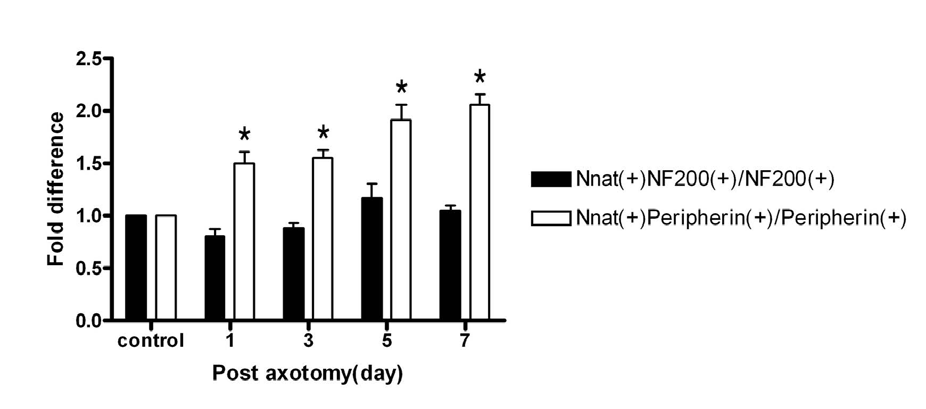
**Statistical analysis of immunohistochemistry of DRG neurons from control and post-axotomy day 1, 3, 5, and 7 rats**. Five to six sections of 10 μm thickness (minimal separation of 90-100 μm) from left L4 or L5 DRG were evaluated. Double immunofluorescence labeling using anti-Nnat, anti-peripherin (marker for unmyelinated C-fiber and thinly myelinated A-δ fiber neurons) and anti-NF200 (marker for large myelinated A-β fiber neurons) antibodies were used to differentiate subpopulation of DRG neurons. **Note**: The percentage counted for double-labeling was based on the following criteria: in Nnat stained-positive neurons, neurons also stained-positive for peripherin (peripherin(+)) or NF200 (NF200(+)). Value are presented as mean ± S.E.M. * Indicates significant difference between control and post-axotomy rats analyzed by one-way ANOVA with Tukey's post-hoc analysis (*P *< 0.05, *n *= 3 for each group).

In parallel studies, the expression of Nnat in DRG neurons was examined by immunofluorescence staining using frozen-cut tissues. Five to six sections of 10 μm thickness (minimal separation of 90-100 μm) from left L4 or L5 DRG (*n *= 3 for each group) were evaluated and analyzed by one-way ANOVA with Tukey's post-hoc analysis. Generally, peripheral sensory DRG neurons are classified as small unmyelinated C-fiber or thinly myelinated Aδ-fiber neurons that transmit signals about thermal and noxious stimuli and large myelinated A-β fiber neurons that transmit information about non-noxious stimuli [5,6]. To examine the expression of Nnat in large versus small DRG neurons, we co-localized Nnat by immunofluorescence staining with peripherin, a marker of small unmyelinated C-fiber and thinly myelinated A-δ fiber neurons, and NF200, a marker of large myelinated A-β fiber neurons [7,8]. Figure 3A and 3B show the double labeling for Nnat and peripherin or NF200 in DRGs in control sham rats, and figure 3C and 3D show double labeling in post-axotomy day 7 rats. Neurons that were double immunoreactive for both Nnat and peripherin (small-sized DRG neurons) were increased in axotomized rats compared to the control. However, those neurons that were double immunoreactive for both Nnat and NF200 (large-sized DRG neurons) were no significantly changed (figure 3). Figure 4 shows statistical analysis of immunohistochemistry of DRG neurons from control and post-axotomy day 1, 3, 5, and 7 rats. In neurons that stained positive for both Nnat and peripherin was 41.05 ± 1.3% in control rats and this percentage was significantly higher in rats of post-axotomy day 1, 3, 5, and 7 (61.26 ± 2.9%, 63.82 ± 2.7%, 78.69 ± 5.6%, and 84.47 ± 2.2% respectively) (*P *< 0.05, *n *= 3 for each group). In contrast, we did not find any significant change between control and experimental group in neurons with double stained-positive for Nnat and NF200. This result indicates that after axotomy of sciatic nerve, the expression of Nnat is mainly located at small-sized DRG neurons, such as small unmyelinated C-fiber and thinly myelinated A-δ fiber neurons.

## Discussion

Neuronatin (Nnat) was initially identified as a gene selectively expressed in neonatal brains, especially in the hindbrain and pituitary gland, and is highly conserved in species such as rat, mouse and human [2,9]. Due to the abundant expression of Nnat mRNA during late fetal and early postnatal periods of mammalian brain development, and downregulation during adulthood and senescence, it was suggested that Nnat may be involved in neonatal neuronal cell differentiation during development [1]. Although Nnat is mainly known to be expressed in the central nervous system, particularly in the developmental brain, it is also expressed in non-neuronal tissues such as the pituitary glands, lungs, adrenal glands, uterus, skeletal muscles, ovaries, adipose tissues, and pancreas [10-14].

Nnat has two alternatively spliced forms: neuronatin-α (Nnat-α) and neuronatin-β (Nnat-β). Both had the same translation start site, open reading frame, stop and termination signals with Nnat-α encoding 81 aa and Nnat-β 54 aa. The only difference between the α and β forms of Nnat was the presence in the α-form of an additional 81 nt sequence encoding 27 aa located in the middle of the coding region and the absence of a small transmembrane domain in β-form, which in normal children and adults is present at only low levels [13,15,16]. Tentatively, based on the cDNA sequences, it is suggested that Nnat-α has three exons, the first containing 72 nt encoding 24 aa, the second containing 81 nt encoding 27 aa, and the third with 90 nt encoding 30 aa. As Nnat-β contains only the first and third exons, it appears to be derived from the α-form by splicing out of the middle exon. The shorter β-form first appears in mid gestation (E11-14), a time when the neural tube has closed and neuroepithelial proliferation and commitment to a neuronal or glial fate are taking place. On the other hand, α-form appears even earlier in gestation (E7-10), and the abundance of the α-form was also increased in mid-gestation. The two Nnat isoforms have distinct patterns of expression during development suggesting that there is differential regulation between the two isoforms and that there are possibly different roles played by the two isoforms during development [2,17]. It appears that the splicing mechanism that generates the β-form becomes active during neurogenesis. It may be speculated that the removal of the middle exon may be important to function of this gene [2]. Nnat has also been identified as an imprinting gene, which is maternally imprinted and there is only expression of the paternal allele mapping to mouse chromosome 2. And during development, Nnat expression is regulated by epigenetic modifications [18-21]. By virtue of its expression pattern during development, Nnat has been postulated to participate in the maintenance of segment identity in the mammalian hindbrain [9].

Recent study conducted by Mzhavia et al. [22] showed that Nnat, an imprinted gene with unknown vascular functions, was up-regulated in both db/db and high-fat diet-fed mouse aortas. Their immunohistochemical staining revealed that Nnat was expressed on the vascular endothelium. To investigate the function of Nnat, they used recombinant adenovirus (Ad-Nnat) to overexpress the Nnat gene in primary human aortic endothelial cells (HAECs) and in the mouse aorta in vivo. Their results showed that overexpression of Nnat in HAECs or in mouse carotid artery would induce increased expression of a panel of nuclear factor-kappaB (NF-κB)-regulated genes, including inflammatory cytokines, chemokines, and cell adhesion molecules. They suggested that this effect of Nnat in both db/db and high-fat diet-fed mouse aortas was mediated through phosphatidylinositol 3-kinase and p38 mitogen-activated protein kinase-dependent activation of NF-κB and might also reflect alterations in the methylation status of endothelial cells.

NF-kappaB is a transcription factor which serves as a transducer between extracellular signals and gene expression. It plays a pivotal role in cell regulation, such as proliferation, immune cell activation, apoptosis, stress responses, differentiation and oncogenic transformation [23]. In most unstimulated cells, NF-kappaB is retained as an inactive form in the cytoplasm by binding to members of the inhibitory factor kappaB (IκB) family. Upon appropriate stimulation, IκB is phosphorylated and degraded, allowing NF-kappaB to translocate to the nucleus where it binds DNA and triggers the transcription of target genes, such as proinflammatory cytokines, chemokines, and cell adhesion molecules. NF-kappaB activation may thus amplify the spinal cord and DRG proinflammatory response, thereby further facilitating pain transmission [23,24]. Recently several studies have shown that activation of NF-kappaB occurs in the DRG and spinal cord, which are both involved in the transmission and processing of nociceptive information leading to pathogenesis of neuropathic pain [25,26]. Furthermore, Yang et al. [27] have demonstrated that activation of NF-kappaB in small diameter sensory neurons of DRG is involved in the growth-related oncogene (GRO/KC; CXCL1)-induced enhancement of potassium currents in both nerve injury and inflammatory pain models. In addition, Liu et al. [28] have identified that spinal long-term potentiation of C-fiber evoked field potentials can be induced by tumor necrosis factor-α in neuropathic rats, which is also NF-kappaB dependent process. Therefore, inhibition of NF-kappaB pathway by different strategies has been used to attenuate chronic pain states in different animal models of neuropathic pain [29-31].

In comparison to our study, it was found that after axotomy of sciatic nerve, there were many novel genes with significant expression changes. Many were down-regulated and others were up-regulated. Among them, Nnat was the one which expression was significantly up-regulated. It is striking that Nnat, an imprinting gene strongly expressed mainly in neonatal developing brain, increased its expression in adult rats DRGs after axotomy of sciatic nerve. Although it is not clear why Nnat expression was increased in DRG cells of axotomized rat, it is possible that a peripheral nerve injury such as axotomy of sciatic nerve, being one of the most used animal models of neuropathic pain, is able to create a "micro-environment" stress strong enough to trigger undergoing epigenetic modifications in Nnat leading to unexpected increased expression in adult DRG cells. In addition, based on the Western blot results, the expression profile of total Nnat (Nnat-α and Nnat-β) was significantly up-regulated. This suggested that the increased expression of total Nnat was induced by peripheral nerve injury. Furthermore, according to the immunohistochemical results, it showed that after the sciatic nerve was axotomized, the subcellular localization of Nnat in DRG was confirmed by peripherin-positive neuronal cells, where the expression of Nnat was also increased. This subpopulation of primary afferent neurons demonstrated a low level of expression of Nnat in the control (Sham group) and suggests that Nnat is expressed mainly on unmyelinated C-fiber and thinly myelinated A-δ fiber neurons. This finding is consistent with the up-regulation of total Nnat protein. Nnat may therefore contribute to neuropathic pain after peripheral nerve injury, given that C-fiber and A-δ fiber are the principal neurons for transmitting information about thermal and noxious stimuli [5,6]. This finding was in accordance with the mechanical paw-withdrawal test, which showed that after the sciatic nerve was transected, the withdrawal latency was decreased significantly, suggesting that the increased noxious input from mechanical stimuli induces hyperalgesia characteristic of neuropathic pain.

Therefore, the scenario we speculate here is that sciatic nerve transection in adult rats might create a stressful micro-environment strong enough to trigger epigenetic modifications on the expression of Nnat, notably increasing it to a significant degree in a selective subpopulation of adult DRG neurons, specifically in sensory C and A-δ fiber neurons. Then, Nnat activates NF-kappaB through phosphatidylinositol 3-kinase and p38 mitogen-activated protein kinase signal pathway. The activation of NF-kappaB might trigger a cascade of pathophysiological processes to occur: 1) The expression of NF-kappaB-related inflammatory mediators would be increased to exert their inflammatory component in the model of neuropathic pain; 2) Among the inflammatory mediators expressed, tumor necrosis factor-alpha (TNF-α) has the ability of persistent reactivation of NF-kappaB forming an amplification loop [24], which might be the initial step for activation of others immune cells in DRG and spinal cord causing persistent inflammatory reaction; 3) The activation of NF-kappaB in small diameter sensory neurons, such as C-fiber and A-δ fiber, might induce pathological channel currents enhancement and/or long-term potentiation that lead to a state of chronic pain. Hence, the peripheral and central sensitization characterized in neuropathic pain might be induced and maintained by the possible processes mentioned above. However, since Nnat is not necessarily the only cause for a specific phenotype of neuropathy, it is possible that other novel imprinted genes may exist in regions not identified by genetic studies that may contribute to the same or distinct phenotype of neuropathy. Therefore, further work is necessary to confirm the speculation we proposed here.

## Conclusions

Our data showed that axotomy of sciatic nerve in adult rats induced an increased expression of Nnat in DRG neurons, specifically in unmyelinated C-fiber and thinly myelinated A-δ fiber neurons, the primary afferent neurons involved in the transmission of noxious stimulus. Using this as a model to demonstrate neuropathic pain, we suggest that the expression of Nnat in the primary sensory neurons correlates well with mechanical hyperalgesia observed in our animals. This is the first observational study characterizing Nnat as having a potential functional role in neuropathic pain.

## Competing interests

The authors declare that they have no competing interests.

## Authors' contributions

KHC designed and performed the experiments, analyzed the data, and drafted the manuscript. CHY co-designed and co-performed the experiments and participated in the analysis of the experimental results. JTC participated in the discussion of the experimental results. CHW and WDS co-performed the experiments. CRL conceived the study, coordinated the implementation of the study and revised the final manuscript. All authors read and approved the final manuscript.

## Supplementary Material

Additional file 1 **Intensity of average values of neuronatin (Nnat) in naïve and axotomy groups**. Changes of mean intensity from triplicate naïve (day 0) against triplicate 3, 7, 21, and 40 day axotomy show significantly higher level of intensity of Nnat (accession number: U08290) in post-axotomy groups compared to the naïve group. The mean intensity ± S.D. of Nnat in each group were: naïve = 1881 ± 88, post-axotomy day 3 = 4167 ± 712, post-axotomy day 7 = 3918 ± 946, post-axotomy day 21 = 3570 ± 723, and post-axotomy day 40 = 3078 ± 477. The mean intensity fold changes of Nnat were expressed as 2.2, 2.0, 1.8, and 1.6 times at time points 3, 7, 21, and 40 days post-axotomy respectively compared to naïve group. * Indicates significant difference between naïve and post-axotomy group analyzed by one-way ANOVA with Tukey's post-hoc analysis (*P *< 0.05, *n *= 15 for each group). Click here for file
