# Supplementary figures and images for: Altered neuronatin expression in the rat dorsal root ganglion after sciatic nerve transection
Source: J Biomed Sci. 2010 May 28;17(1):41. doi: 10.1186/1423-0127-17-41 (PMC2894761; doi:10.1186/1423-0127-17-41)

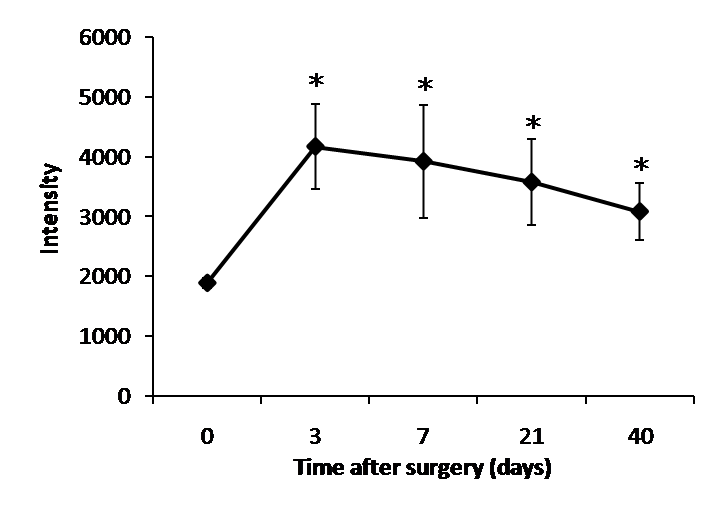

Supplement: Additional file 1 — Intensity of average values of neuronatin (Nnat) in naïve and axotomy groups. Changes of mean intensity from triplicate naïve (day 0) against triplicate 3, 7, 21, and 40 day axotomy show significantly higher level of intensity of Nnat (accession number: U08290) in post-axotomy groups compared to the naïve group. The mean intensity ± S.D. of Nnat in each group were: naïve = 1881 ± 88, post-axotomy day 3 = 4167 ± 712, post-axotomy day 7 = 3918 ± 946, post-axotomy day 21 = 3570 ± 723, and post-axotomy day 40 = 3078 ± 477. The mean intensity fold changes of Nnat were expressed as 2.2, 2.0, 1.8, and 1.6 times at time points 3, 7, 21, and 40 days post-axotomy respectively compared to naïve group. * Indicates significant difference between naïve and post-axotomy group analyzed by one-way ANOVA with Tukey's post-hoc analysis (P < 0.05, n = 15 for each group). [file 1423-0127-17-41-S1.TIFF]
